# Coho salmon spawner mortality in western US urban watersheds: bioinfiltration prevents lethal storm water impacts

**DOI:** 10.1111/1365-2664.12534

**Published:** 2015-10-08

**Authors:** Julann A. Spromberg, David H. Baldwin, Steven E. Damm, Jenifer K. McIntyre, Michael Huff, Catherine A. Sloan, Bernadita F. Anulacion, Jay W. Davis, Nathaniel L. Scholz

**Affiliations:** ^1^ Ocean Associates, Under Contract to Northwest Fisheries Science Center National Marine Fisheries Service NOAA 2725 Montlake Blvd. E. Seattle WA 98112 USA; ^2^ Environmental and Fisheries Science Division Northwest Fisheries Science Center National Marine Fisheries Service NOAA 2725 Montlake Blvd. E. Seattle WA 98112 USA; ^3^ U.S. Fish and Wildlife Service Washington Fish and Wildlife Office 510 Desmond Dr. S.E. Lacey WA 98503 USA; ^4^ Puyallup Research and Extension Center Washington State University 2606 W. Pioneer Ave. Puyallup WA 98371 USA; ^5^ Suquamish Tribe PO Box 498 18490, Suquamish Way Suquamish WA 98392 USA

**Keywords:** habitat restoration, non‐point source pollution, Pacific salmon, run‐off, storm water, urban ecology, urban streams

## Abstract

1. Adult coho salmon *Oncorhynchus kisutch* return each autumn to freshwater spawning habitats throughout western North America. The migration coincides with increasing seasonal rainfall, which in turn increases storm water run‐off, particularly in urban watersheds with extensive impervious land cover. Previous field assessments in urban stream networks have shown that adult coho are dying prematurely at high rates (>50%). Despite significant management concerns for the long‐term conservation of threatened wild coho populations, a causal role for toxic run‐off in the mortality syndrome has not been demonstrated.
2. We exposed otherwise healthy coho spawners to: (i) artificial storm water containing mixtures of metals and petroleum hydrocarbons, at or above concentrations previously measured in urban run‐off; (ii) undiluted storm water collected from a high traffic volume urban arterial road (i.e. highway run‐off); and (iii) highway run‐off that was first pre‐treated via bioinfiltration through experimental soil columns to remove pollutants.
3. We find that mixtures of metals and petroleum hydrocarbons – conventional toxic constituents in urban storm water – are not sufficient to cause the spawner mortality syndrome. By contrast, untreated highway run‐off collected during nine distinct storm events was universally lethal to adult coho relative to unexposed controls. Lastly, the mortality syndrome was prevented when highway run‐off was pretreated by soil infiltration, a conventional green storm water infrastructure technology.
4. Our results are the first direct evidence that: (i) toxic run‐off is killing adult coho in urban watersheds, and (ii) inexpensive mitigation measures can improve water quality and promote salmon survival.
5. *Synthesis and applications*. Coho salmon, an iconic species with exceptional economic and cultural significance, are an ecological sentinel for the harmful effects of untreated urban run‐off. Wild coho populations cannot withstand the high rates of mortality that are now regularly occurring in urban spawning habitats. Green storm water infrastructure or similar pollution prevention methods should be incorporated to the maximal extent practicable, at the watershed scale, for all future development and redevelopment projects, particularly those involving transportation infrastructure.

## Abstract 2

Coho salmon, an iconic species with exceptional economic and cultural significance, are an ecological sentinel for the harmful effects of untreated urban run‐off. Wild coho populations cannot withstand the high rates of mortality that are now regularly occurring in urban spawning habitats. Green storm water infrastructure or similar pollution prevention methods should be incorporated to the maximal extent practicable, at the watershed scale, for all future development and redevelopment projects, particularly those involving transportation infrastructure.

Editor's Choice

## Introduction

In recent decades, non‐point source run‐off has become the leading pollution threat to aquatic habitats in the USA and similarly developed countries. In highly built watersheds, the transport of toxic chemical contaminants via storm water contributes to the well documented ‘urban stream syndrome’, as evidenced by various indicators of biological and ecological degradation (Walsh *et al*. 2005). These include declines in species abundance, species diversity and the proliferation of non‐native, pollution‐tolerant taxa.

Nevertheless, field assessments in urban watersheds rarely report fish kills or similar acute mortality events for aquatic life. A notable exception is the recurring die‐off of adult coho salmon that return from the ocean to spawn each year in large metropolitan areas of northern California, western Oregon and Washington in the USA, and southern British Columbia in Canada. The coho mortality phenomenon has been studied most extensively in lowland streams of the greater Seattle area of Puget Sound. Coho begin the freshwater phase of their spawning migration with the onset of autumn rainfall. Typically within days of arriving at stream reaches suitable for spawning, affected fish become stricken with symptoms that progress from a loss of orientation (surface swimming) to a loss of equilibrium and death on a time‐scale of a few hours (Videos S1 and S2, Supporting information; Scholz *et al*. 2011). Year‐to‐year mortality rates within and across urban watersheds are typically high (~50–90%), as measured by the proportion of unspawned females for an entire annual run (Scholz *et al*. 2011).

As might be expected, initial modelling indicates that such high mortality rates at the critical spawner life stages pose a significant extinction risk for wild coho populations (Spromberg & Scholz 2011). Coho distinct population segments, or evolutionarily significant units (ESUs; Waples 1991), are comprised of metapopulations that span large river basins with varying degrees of urban and suburban land use (e.g. Pess *et al*. 2002; Bilby & Mollot 2008). This population structure and the highly migratory life histories of salmonids have generally constrained ecotoxicological studies (Ross *et al*. 2013). Nevertheless, if urban run‐off is killing adult coho, ongoing regional development pressures may present an important obstacle to the recovery of coho ESUs, including those designated as threated (Lower Columbia River) or a species of concern (Puget Sound) under the US Endangered Species Act.

To date, the evidence linking urban storm water run‐off and coho spawner mortality has been indirect. The uniform nature of the symptoms, over many years and across many streams, is consistent with a common and prevalent form of toxicity. A forensic investigation spanning nearly a decade ruled out several other potential causes, including conventional water quality parameters (e.g. dissolved oxygen, temperature), habitat availability, poor spawner condition and disease (Scholz *et al*. 2011). Moreover, an initial geospatial land cover analysis found a significant positive association between the severity of the coho die‐off phenomenon and the extent of impervious surface within a watershed (Feist *et al*. 2011).

The aim of the present study was to explore the connection between water quality and coho mortality more directly by experimentally exposing freshwater‐phase spawners to both artificial and actual highway run‐off. Although urban storm water is chemically complex, field collected samples consistently contain motor vehicle‐derived mixtures of metals and polycyclic aromatic hydrocarbons (PAHs), many of which are toxic to salmon at other life stages (e.g. copper, McIntyre *et al*. 2012; Sandahl *et al*. 2007; PAHs, Meador *et al*. 2006; Heintz *et al*. 2000). If the mortality syndrome could be reproduced with an environmentally realistic mixture of metals and PAHs, it would then be possible to identify the causal agents by removing different components of the mixture. To account for the possibility that some other contaminant(s) may be causal, we also exposed adult coho to storm water collected from a dense urban arterial road (i.e. highway run‐off). Lastly, we exposed adult coho to highway run‐off which was pre‐treated with a conventional green storm water infrastructure (GSI) technology (bioinfiltration through soil columns) to remove pollutants, with the aim of lessening or eliminating any overtly harmful impacts of unmitigated storm water.

## Materials and methods

### Animals

Adult coho salmon were collected at the Suquamish Tribe's Grovers Creek Hatchery near Poulsbo, Washington. Hatchery coho are an appropriate surrogate for wild coho given that field observations have documented the mortality syndrome in spawners of both wild and hatchery origins (Scholz *et al*. 2011). At Grovers Creek, returning coho migrate <4 km in freshwater from Miller Bay in Puget Sound to a hatchery pond via a fish ladder. The pond was seined on Monday, Wednesday and Friday of each week, and thus the fish were in the pond for a maximum of 72 h prior to capture. The coho were strays from a net‐pen operation designed to provide a terminal fishery to the south of Miller Bay. When available, females were used for the controlled storm water exposures. For trials with an insufficient number of females, males were also included, as the urban mortality syndrome affects males and females alike (Scholz *et al*. 2011). Only fish exhibiting normal behaviour and with no obvious signs of trauma, disease or poor condition were included. One set of exposures was conducted on a given day.

Each individual coho spawner was placed in a holding tube constructed of PVC, of either 15·2 × 76·2 cm (diameter × length) or 20·3 × 106·7 cm with 1·1‐cm‐thick polyethylene gates fitted into slots at either end. Ventilation was provided by six 2·5‐cm‐diameter holes on either side of the anterior (head) end of each tube and five 1·75‐cm‐diameter holes in each gate. A ventilation hose attached to a pump (for 2011–12, a Flotec Tempest 1/6 HP, 4·5 m^3^ h^−1^ (Flotec Water, Delavan, WI, USA); for 2013–14, a Lifegard Aquatics Quiet One 3000, 3·1 m^3^ h^−1^ (Lifegard Aquatics, Cerritos, CA, USA)) submerged in the polyethylene tank supplied a minimum of 4 L min^−1^ flow through the forward gate and over each fish in an anterior–posterior direction.

For each trial, four separate coho holding tubes were placed in a large polyethylene tank containing 440 L of clean well water, artificial storm water, highway run‐off, or run‐off pretreated with soil infiltration. Adult coho were exposed for 4–48 h depending on the treatment (see below). Aeration was provided with air stones attached to an air pump (Coralife 05146 Model SL‐38 Super Luft Air Pump, Central Aquatics‐Coralife, Franklin, WI, USA). Exposure waters were maintained at temperatures below 14 °C by flow‐through (2011) and Aqua Logic^®^ Cyclone^®^ Drop‐In Titanium Chillers (2012). Smaller ventilation pumps that produced less heat were used in 2013–14, and thus, chillers were not needed.

### Exposures to Artificial Storm Water

In the autumn of 2011, returning adult coho were exposed to artificial storm water containing mixtures of PAHs and metals. The mixtures were comprised of individual compounds at concentrations at or above those measured during autumn storm events in Seattle‐area urban streams (Seattle Public Utilities 2007), or at levels representative of urban storm water run‐off more generally (Stein, Tiefenthaler & Schiff 2006; Gobel, Dierkes & Coldewey 2007; Tiefenthaler, Stein & Schiff 2008). The PAH profile of urban run‐off is compositionally similar to that of crude oil, particularly for toxic three‐ and four‐ring compounds (McIntyre *et al*. 2014). The exception is a lack of dissolved pyrene and fluoranthene in crude oil (Incardona *et al*. 2009). Thus, the PAH portion of the mixture was generated from a water‐accommodated fraction (WAF) of Alaska North Slope crude oil, to which pyrogenic pyrene and fluoranthene were added (Table S1). The WAFs were prepared in a 3‐speed commercial blender with a 3·8‐L stainless steel container (Waring CB15; Waring Commercial, Torrington, CT, USA), following a protocol developed to yield fine oil droplets and bioavailable PAHs in the dissolved phase (Incardona *et al*. 2013). In brief, the stainless steel container was cleaned with acetone and dichloromethane, the rubber lid was lined with dichloromethane‐rinsed heavy‐duty aluminium foil, and the container was filled with 1 L of deionized water. The volume of crude oil added to the WAF (1 mL) was intended to produce a final maximum phenanthrene exposure concentration of 0·384 μg L^−1^ (Stein, Tiefenthaler & Schiff 2006). Pyrene and fluoranthene were then added to coequal final target exposure concentrations of 0·584 μg L^−1^. Water and oil were blended for 30 s on the lowest speed four times. The oil–water mixture was then poured into a 1‐L separatory funnel and allowed to sit for 1 h. With care to leave the surface slick undisturbed, 789·3 mL at the bottom were then drawn off and added to the exposure chamber.

The metals fraction of the PAHs/metals mixture consisted of cadmium, nickel, lead, copper and zinc (anhydrous CdCl_2_, NiCl_2,_ PbCl_2,_ CuCl_2_ and ZnCl_2_; Sigma‐Aldrich, St. Louis, MO, USA, > 98% purity) added to clean well water at nominal concentrations (Table S1) that were in the upper range of metal detections in urban streams (Stein, Tiefenthaler & Schiff 2006; Gobel, Dierkes & Coldewey 2007; Seattle Public Utilities 2007; Tiefenthaler, Stein & Schiff 2008). Moreover, the concentrations of metals in urban run‐off are transiently elevated during the first flush interval (Kayhanian *et al*. 2012). To capture this exposure scenario, experiments in the autumn of 2012 used relatively higher nominal concentrations of metals only (Table S1). Temperature and dissolved oxygen were monitored and maintained at physiological ranges for adult salmon, and water samples were collected for analytical verification of exposure concentrations.

### Exposures to Highway Run‐Off

Storm water was collected from the downspouts of an elevated urban principal arterial road in Seattle, WA. The downspouts receive run‐off from the on‐ramp to a four‐lane (70 m wide) highway over which approximately 60 000 motor vehicles travel each day (WA DOT 2013a). The highway, paved with Portland cement concrete (WA DOT 2013b), is a conventional urban impervious surface. All of the flow to the downspouts originated from precipitation falling on the active arterial road.

**Figure 1:**
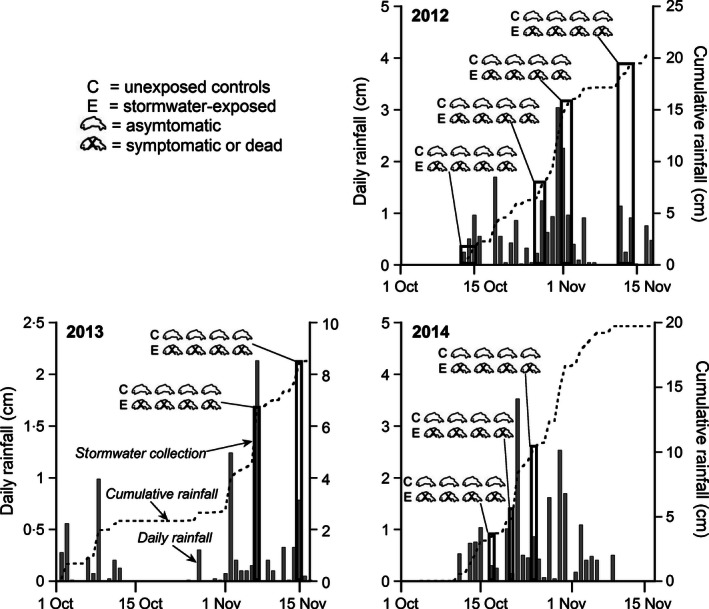
The presence or absence of the pre‐spawn mortality syndrome in adult coho salmon exposed to unfiltered highway run‐off (E) or clean well water (C). Paired exposures spanned three consecutive autumn spawning seasons, 2012–14. Shown in each panel are daily rainfall (shaded bars), cumulative rainfall (dotted lines), highway run‐off collection intervals for each separate exposure event (black rectangles) and the presence or absence of symptomatic (or dead) fish in each individual treatment (4–24 h duration; see Materials and methods). Symptoms included lethargy, loss of orientation or loss of equilibrium.

The captured run‐off was transported to the hatchery facility in either covered glass carboys or in a stainless steel tank. The holding interval prior to exposures varied with the timing and intensity of autumn storm events, but did not exceed 72 h. While some collections took place after an extended antecedent dry interval and therefore included the first flush for a given storm, others spanned periods of intermittent rainfall. Daily and cumulative rainfall for each autumn season is shown in Fig. 1, with each storm water collection interval superimposed (solid rectangular boxes). Collected run‐off was used for one exposure only. Temperature, pH and dissolved oxygen were measured at the outset of each exposure, and water samples were collected for chemical analyses to quantify concentrations of metals and PAHs (2012–13 but not 2014 storms). After exposures, the run‐off was transported to a Kitsap County Wastewater Pump Station for disposal.

### Exposures to Filtered Run‐Off

In the autumn of 2013, four 200‐L bioretention columns were constructed and plumbed with outflow drains following conventional guidelines for green storm water infrastructure (WA DOE 2012). The filtration columns were composed of a 30·5‐cm drainage layer of gravel aggregate overlain by 61 cm of bioretention soil media (60% sand: 40% compost) and topped with 5 cm of mulched bark. In the autumn of 2014, the bioretention columns were emptied and fresh media installed. In each year, the bioretention media were conditioned by passing seven pore volumes (660 L) of clean well water from the hatchery facility through each column at a rate of 2 L min^−1^: equivalent to 2 months of summer rainfall on a contributing area 20 times the size of the treatment area. Urban highway run‐off was collected as described above, and the homogenized volume was evenly divided to flow through one of the four bioretention columns at a rate of 2 L min^−1^, with the outflows from the four columns recombined into a single post‐treatment exposure volume. Adult coho spawners were exposed to either untreated urban storm water or the same run‐off post‐filtration for 4–24 h. Water quality was measured, and samples were collected for chemical analyses as described above.

### Observations of Symptomatic Fish

Hallmark characteristics of the adult coho spawner mortality syndrome include a progression from lethargy to a loss of orientation, a loss of equilibrium, followed by death (Scholz *et al*. 2011). Individual fish were examined for these symptoms during and after each exposure. Fish were moved from the large exposure tank and released from their holding tubes into an observation tank containing clean well water at a minimum depth of 50 cm. Swimming ability and evasiveness (responses to light and gentle prodding) were recorded over a 1‐ to 3‐min observation interval. For the trials using artificial storm water, symptomology was assessed at 24 h and then again at the end of the exposure. Coho exposed to highway run‐off were visually examined at 2, 4 and 24 h. Live fish at 2 and 4 h were returned to their holding tubes and exposure chambers for the remainder of the trial.

### Water Quality Analyses

Conventional water quality parameters, including pH, dissolved oxygen, alkalinity, total suspended solids, N‐ammonia, nutrients and organic carbon, were measured for selected trails using standard instrumentation or by outside laboratories using US EPA‐approved methods (Analytical Resources Inc., Tukwila, WA, USA or Am Test Inc., Kirkland, WA, USA). Total and dissolved concentrations of cadmium, copper, nickel, lead and zinc were determined by inductively coupled plasma mass spectrometry (ICP‐MS) by Frontier Global Sciences (Bothell, WA, USA; EPA method 1638) or Am Test Inc. (EPA method 200.8). Briefly, samples were preserved in 1% (v/v) nitric acid (total metals) or passed through a 0·45‐μm filter (dissolved metals) and then oven‐digested prior to analysis by ICP‐MS. Duplicate samples and laboratory blanks were included to ensure quality control. Selected water samples for PAH determinations were preserved with 10% dichloromethane and stored at 4 °C in amber glass bottles until analysis at the NOAA Northwest Fisheries Science Center by gas chromatography/mass spectrometry (GC‐MS) with additional selected ion monitoring for alkyl‐PAHs (Sloan *et al*. 2014).

### Tissue Sampling and Analyses

At the conclusion of each exposure, fish length, weight, reproductive status and origin (i.e. hatchery or wild spawned) were assessed. To confirm the bioavailability of PAHs in exposure waters, bile was screened for PAH metabolites in a subset of 2011 and 2012 trials with both artificial storm water and highway run‐off. Fish were killed, and bile was collected from the gall bladder and stored in amber glass vials at −20 °C until analysis for PAH metabolites using high‐performance liquid chromatography with fluorescence detection (Krahn *et al*. 1986; da Silva *et al*. 2006). The concentrations of fluorescent PAH metabolites in bile are determined using naphthalene (NPH), phenanthrene (PHN) and benzo[a]pyrene (BaP) as external standards and converting the relative fluorescence response of bile to NPH, PHN and BaP equivalents, and reported as ng g^−1^ bile or ng mg^−1^ biliary protein.

Coho gills were sampled to confirm uptake of metals in selected 2011 and 2012 artificial and collected storm water exposures. Tissues were excised with Teflon or titanium scissors and plastic forceps, placed in plastic Whirl‐paks, and stored at −80 °C. Metals analyses were determined by inductively coupled mass spectroscopy (ICP‐MS) at the Trace Elements Research Laboratory (TERL; College Station, TX, USA) using standard methods (TERL Method Codes 001, 006). Briefly, gill tissues were wet digested with nitric acid, freeze‐dried, and homogenized by ball‐milling in plastic containers. Samples were ionized in high‐temperature argon plasma, and positively charged ions were separated on the basis of their mass : charge ratios by a quadrupole mass spectrometer. Student's *t*‐tests assessed differences in the tissue concentrations between exposures and their respective paired control.

## Results

### Adult Coho Responses Across Treatments

Coho spawners exposed for 24 h to mixtures of PAHs and metals at concentrations slightly higher than those previously measured in urban run‐off were asymptomatic – that is behaviourally indistinguishable from controls exposed to clean well water (Table 1). Although there was some mortality across the four independent trials (*n* = 4 of 30 fish total), this was not significantly different by treatment (Fisher exact tests, two‐tailed, *P *≥* *0·21) and was therefore apparently attributable to handling stress. Extending the exposures to 48 h did not increase the incidence of mortality or symptomology (*n* = 4 of 22 fish total, two control and two exposed). Increasing the concentrations of metals fivefold or 10‐fold in metal‐only mixtures was also insufficient to elicit the symptoms of the pre‐spawn mortality syndrome (Table 2). As with the PAHs/metals mixture, there was a small but insignificant amount of mortality across treatments (*n* = 2 of 38 fish; Fisher exact tests, two‐tailed, *P *=* *1).

**Table 1:** Adult coho salmon spawner mortality following a 24‐h exposure to either clean well water (unexposed) or a mixture of polycyclic aromatic hydrocarbons (PAHs) and metals. Shown in parentheses are the numbers of symptomatic or dead fish as a proportion of the total numbers of spawners in each exposure. The PAH/metal exposures were based on measured levels in urban creeks during storm events (see Materials and methods). Relative to environmental samples, the artificial mixture contained higher concentrations of both total PAHs and metals. Each exposure was conducted on a separate day

Exposure (h)	Mortality	
	Unexposed	PAHs/Metals mixture
24	25% (1/4)	0% (0/4)
24	33% (1/3)	0% (0/3)
24	0% (0/4)	50% (2/4)
24	0% (0/4)	0% (0/4)

**Table 2:** Exposures to relatively high levels of metals in artificial mixtures are not sufficient to elicit the coho spawner mortality syndrome. Similar to unexposed controls, nearly all of the adults survived exposures to mixtures of metals (Cd, Cu, Pb, Ni, Zn) that were fivefold (Low) or 10‐fold (High) higher than measured concentrations in urban creeks where coho mortality syndrome was observed. Shown in parentheses are the numbers of symptomatic or dead fish as a proportion of the total numbers of spawners in each exposure. Each exposure was conducted on a separate day

Exposure (h)	Mortality		
	Unexposed	Low metals	High metals
24	0% (0/4)	0% (0/4)	
24	0% (0/4)	0% (0/3)	
24	0% (0/4)		0% (0/4)
24	25% (1/4)		25% (1/4)
24	0% (0/3)		0% (0/4)

Although the artificial storm water preparations were designed to have a similar composition to highway run‐off for many PAHs and metals, the effects on coho spawners were very different. Whereas the artificial mixtures did not elicit the distress characteristic of the mortality syndrome, coho exposed to the unfiltered highway run‐off rapidly became symptomatic. For every discrete rainfall collection interval (*n* = 9; 2012–2014), all of the exposed fish were either symptomatic or dead within 4 h (Fig. 1, Table 3). Those that survived the initial 4‐h exposure were dead by 24 h. All of the paired control coho in clean well water survived, showing no behavioural symptoms at 4 or 24 h (Fig. 1, Table 3). Each exposure showed a statistically significant difference in mortality (Fisher exact tests, two‐tailed, *P *=* *0·006). Examples of asymptomatic control fish and symptomatic, run‐off‐exposed spawners are shown in Video S3. For the purpose of comparing symptoms, digital movies of affected coho in Seattle‐area urban watersheds are shown in Videos S1 and S2. Thus, despite the event‐to‐event variation in rainfall duration and intensity, and a corresponding variation in water chemistry (conventionals, metals and PAHs, Tables S2, S3 and S5), urban run‐off was 100% lethal to otherwise healthy adult coho salmon. The contribution of handling stress was evidently minimal, as the survival rate for controls across treatments in 2012–2014 was 100%.

**Table 3:** Proportion of adult coho displaying the spawner mortality syndrome after placement in clean well water (unexposed) or highway run‐off that was either unfiltered or filtered through an experimental soil bioretention system (during 2013 and 2014). Shown in parentheses are the numbers of symptomatic or dead fish as a fraction of the total number of coho in each treatment. Each exposure was conducted on a separate day

Exposure (h)	Mortality		
	Unexposed	Unfiltered	Filtered
4	0% (0/4)	100% (4/4)	0% (0/4)
24	0% (0/4)	100% (4/4)	0% (0/4)
24	0% (0/4)	100% (4/4)	0% (0/4)
24	0% (0/4)	100% (4/4)	0% (0/4)
24	0% (0/4)	100% (4/4)	0% (0/4)

The constructed bioretention columns effectively treated the highway run‐off in terms of both toxic chemical exposure and salmon spawner survival. Although the focal (measured) contaminants were not completely removed by infiltration, the overall improvement in water quality was sufficient to completely prevent the lethal effects and sublethal symptomology caused by untreated storm water. All of the adult coho exposed to filtered run‐off survived and showed no behavioural symptoms at either 4 or 24 h (100% survival, *n* = 20; Table 3; Video S3). Thus, urban storm water contains an as‐yet unidentified chemical component(s) that, while lethal to salmon spawners, can be removed using inexpensive bioinfiltration.

### Measured Levels of Metals, PAHs and Conventional Water Quality Parameters Across Treatments

The chemical properties of highway run‐off were evaluated for the six distinct collection events in the autumn of 2012 and 2013. As expected, conventional water quality parameters varied across storm water collections, as did concentrations of PAHs and metals. The analytical results are shown in Tables S2, S3 and S5. As expected, suspended solids (TSS: 23–220 mg L^−1^) and organic matter (DOC: 8–92 mg L^−1^) were elevated in urban run‐off relative to control water (TSS < 1·1 mg L^−1^, DOC < 1·8 mg L^−1^). In contrast, run‐off had lower Mg (*t*(8)* *=* *6·072, *P *<* *0·001), alkalinity (*t*(8)* *=* *6·201, *P *<* *0·001) and phosphate (*t*(8)* *=* *3·547, *P *=* *0·008). The pH values for run‐off were circumneutral (6·12–7·47) and consistently lower than those for control water (*t*(8)* *=* *2·691, *P *=* *0·027). Other conventional chemistry parameters were not significantly different among treatments, including Ca (*t*(8)* *=* *−0·121, *P *=* *0·907) and hardness (*t*(8)* *=* *1·159, *P *=* *0·280). At the outset of exposures, dissolved oxygen levels ranged from 8·1 to 10·7 mg O_2_ L^−1^ and were maintained above 6·5 mg L^−1^ with additional aeration as needed.

**Figure 2:**
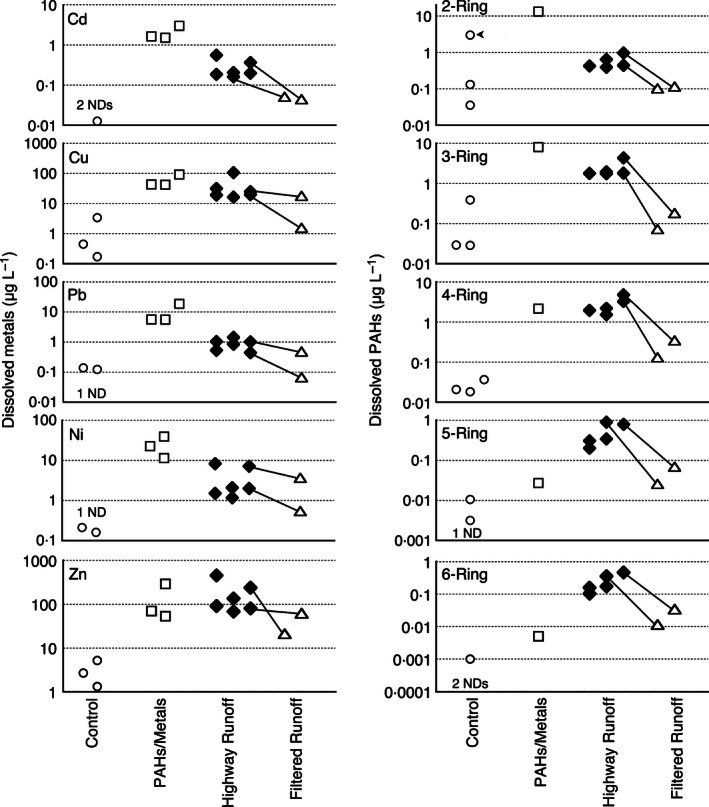
Dissolved metal (left column) and dissolved polycyclic aromatic hydrocarbon (right column) concentrations summarized by ring number for adult exposures to well water controls, polycyclic aromatic hydrocarbons (PAHs)/metal mixtures, highway run‐off and filtered run‐off. Closed symbols indicate dead or symptomatic individuals were observed in the exposure. Lines connect paired highway run‐off and filtered run‐off from the same collection. Control points are the mean of samples collected each year. The number of mean values below the reporting limits (i.e. non‐detects) is indicated by # ND.

**Figure 3:**
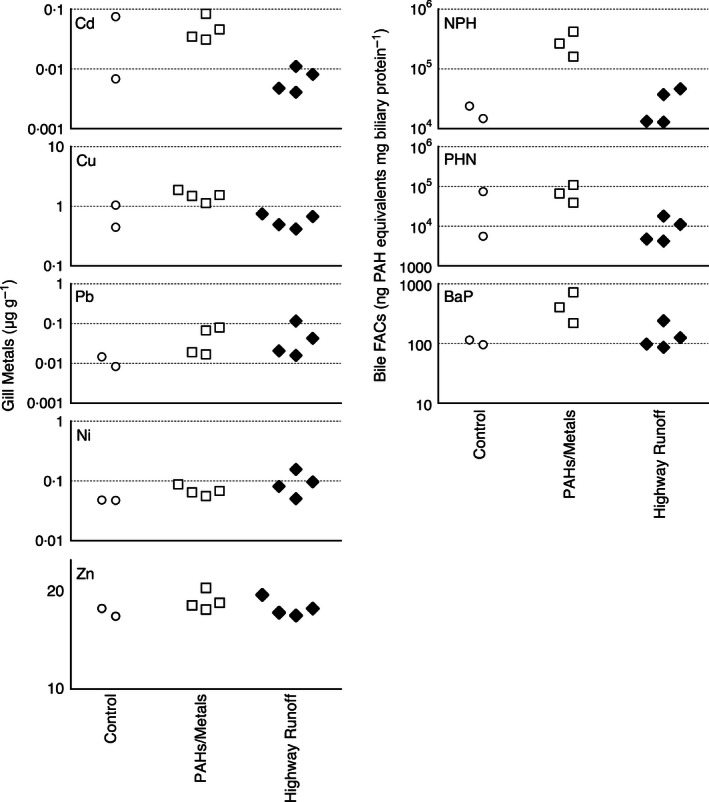
Left column shows the relative measured concentrations of metals in adult coho salmon gill tissue for Cd, Cu, Pb, Ni and Zn (μg g^−1^). Control values are means of control tests run in 2011 and 2012. Closed symbols indicate dead or symptomatic individuals observed in the exposure. The right column shows bile fluorescent aromatic compounds (FACs) detected at naphthalene (NPH), phenanthrene (PHN), benzo‐a‐pyrene (BAP) wavelengths shown as protein corrected polycyclic aromatic hydrocarbons (PAH) equivalents (ng mg^−1^).

Collected highway run‐off had a more pyrogenic (or combustion‐driven) PAH profile relative to the artificial storm water mixtures, as evidenced by a relative enrichment of higher molecular weight (5‐ and 6‐ring) compounds and fewer low molecular weight (2‐ and 3‐ring) compounds (Fig. 2). Bile PAH metabolites were not significantly different between fish exposed to control well water or storm water run‐off after a 4‐h exposure (Fig. 3). Although the measured concentrations of PAH metabolites in the bile of fish exposed for 24 h to the PAHs/metals mixture were elevated relative to paired controls, the difference was not significant (Student's *t*‐test; *P *=* *0·1, 0·14, 0·11 for phenanthrene, benzo‐a‐pyrene and naphthalene metabolites, respectively). This indicates that low‐level PAH exposures typical of urban run‐off do not produce large increases in measurable bile metabolites, consistent with bile PAH metabolite measurements from symptomatic coho previously collected during field surveys of urban spawning habitats (Scholz *et al*. 2011).

Notably, in 2012, the levels of 2‐ and 3‐ring PAHs in the control exposure water were unexpectedly elevated relative to all other control treatments (Fig. 2, arrow). This was attributed to the recent drilling of a new well at the Suquamish hatchery facility. Measured PAH levels in the well water declined sharply over a time span of 2 weeks (Fig. 2), and adult coho controls that were exposed during the interval did not exhibit behavioural symptoms (Fig. 1).

Whereas the levels of dissolved‐phase Cd and Pb were generally lower in collected run‐off relative to all of the artificial storm water mixtures (Fig. 2), Cu and Ni in run‐off spanned the range of these two metals in the environmentally relevant mixture. Zinc levels in run‐off were higher, and within the range of corresponding Zn levels in the high‐metal mixture. The concentrations of metals in the gills of storm water‐exposed and unexposed coho (4 h) were not significantly different (Student's *t*‐tests, *P *>* *0·05; Fig. 3) and, in both cases, were lower than gill metal levels measured from symptomatic spawners collected from the field (Scholz *et al*. 2011). Similarly, exposures to the environmentally relevant artificial storm water mixture of PAHs/metals did not produce a significant accumulation of metals in the gills, with the exception of Ni (Student's *t*‐test, *P *=* *0·017). For the high metals mixture, only gill Cd, Cu and Pb levels were significantly elevated relative to controls (Student's *t*‐test; *P *=* *0·002, 0·018, 0·003 for Cd, Cu and Pb, respectively).

Filtering collected highway run‐off through the bioretention columns reduced total PAHs by 94% and total metals by 58%. As expected, removal efficiency varied for different contaminants. For example, the soil columns removed lower molecular weight PAHs less efficiently than higher molecular weight PAHs (e.g. 81–89% for 2–3 ring PAHs vs. 93% removal of 4‐ to 5‐ring PAHs; Table S5). Notably, the medium in the bioretention columns was a source (i.e. an exporter) of total Ni to the treated run‐off, resulting in a 57% increase over the pre‐filtration input (Table S3). All other total metals decreased by an average of 48–88% across the two events in the order of Cd < Pb < Cu < Zn. For each of the metals, concentrations in the dissolved phase also declined after soil column infiltration (Table S3). In addition to exporting Ni, the bioretention columns were also a source of DOC (post‐/pre‐filtration increase of 164%), alkalinity (+29%), Ca (+60%), Mg (+372%), ortho‐P (+4000%) and increasing hardness (+107%). By contrast, column infiltration reduced the ammonia content of storm water by 92% (Table S2).

## Discussion

We have confirmed that controlled exposures to untreated urban run‐off are sufficient to reproduce the coho spawner mortality syndrome. Adult coho became symptomatic and died within a few hours of immersion in collected storm water. Mortality rates were 100% for exposed fish vs. 0% in control fish held in clean well water, and these results were consistent across nine distinct rainfall intervals that spanned three consecutive autumn spawning runs. As evidence that one or more toxic chemical contaminants are causal, pre‐treating the highway run‐off with soil bioinfiltration completely prevented the acutely lethal impacts on coho spawners. Surprisingly, coho did not develop symptoms in response to artificial mixtures of PAHs and metals, even at concentrations that were higher than those typically measured in storm water, including the first flush. Urban run‐off is chemically complex, with many chemical constituents that are very poorly characterized in terms of toxicity to fish. While it may take years of additional assessment to identify precisely which of these agents is killing coho, our initial results suggest that simple GSI technologies hold promise as a means to improve water quality and effectively prevent coho mortality in urban spawning habitats.

Our finding that road run‐off alone is sufficient to induce the spawner mortality syndrome aligns with previous evidence for a positive association between the amount of impervious surface within an urban watershed and the year‐to‐year severity of coho die‐offs (Feist *et al*. 2011). It appears that other forms of water quality degradation are not necessary to produce the phenomenon. Consistent with this, symptomatic spawners do not show evidence of neurotoxic pesticide exposure (Scholz *et al*. 2011), and adult coho are not unusually vulnerable to low‐level mixtures of currently used pesticides (King *et al*. 2013). The link to impervious run‐off also discounts a role for personal care products, pharmaceuticals, and other classes of compounds that are transported to some urban streams via combined sewer overflows in heavy rains.

As noted above, urban road run‐off contains a complex mixture of chemicals, many of which originate from motor vehicles in the form of exhaust, leaking crankcase oil and the wearing of friction materials (i.e. brake pads) and tyres. We assessed the toxicity of PAH and metal mixtures because these compounds are ubiquitous in storm water and are known to be disruptive to the fish cardiovascular system (PAHs: Brette *et al*. 2014), as well as the respiratory and osmoregulatory functions of the gill (metals: Niyogi & Wood 2004). Although bile and gill tissue results suggest that PAHs and some metals are bioavailable to the coho spawners (this study; Scholz *et al*. 2011), artificial mixtures of PAHs and metals did not produce the symptoms of the mortality syndrome. Our results appear to rule out many of the PAHs that are common to urban run‐off and crude oil spills (e.g. phenanthrenes). However, there may be a role for the higher molecular weight pyrogenic PAHs found in particulate vehicle exhaust (i.e. soot), other than pyrene or fluoranthene. The remaining list of potential causal chemicals is long and includes other organic hydrocarbons such as methylphenols, quinones, thiazoles, thiophenes, furans and quinolines. Given the logistical challenges associated with adult coho exposures – seasonal availability of animals, large volume assays, limited number of fish, etc. – it may be years before the causal agent(s) is identified. Notably from a water resource management perspective, this will likely be a chemical or chemicals for which there are no existing water quality criteria.

Biological indicators play an important role in field assessments to document the urban stream syndrome in affected watersheds world‐wide. Common examples are benthic indices of biological integrity (B‐IBIs), which are used to characterize the health of streams based on the diversity and abundance of macroinvertebrates (Karr 1999). Although poor B‐IBI scores are diagnostic of aquatic habitat degradation, they do not necessarily differentiate between drivers that may be chemical (i.e. pollution) vs. physical or biological. Conversely, biological indicators that are specific to toxic run‐off may not have directly meaningful implications for individual survival, as a basis for guiding species conservation at the population and community scales. This includes, for example, the upregulation of sensitive and responsive cytochrome p450 enzymes in the livers of fish exposed *in situ* to certain PAHs and other contaminants that act via the aryl hydrocarbon receptor (van der Oost, Beyer & Vermeulen 2003).

Coho spawners, by contrast, appear to be very sensitive ecological indicators, with a response metric that is directly attributable to toxic storm water. Moreover, the implications of widespread and recurring mortality are relatively clear at higher scales (e.g. Spromberg & Scholz 2011). Although the highway run‐off used in this study (at the point of discharge) presumably contained higher concentrations of chemical contaminants than surface water conditions in urban spawning habitats, it is evident that run‐off in urban waterways is not sufficiently diluted to protect many or most coho from premature death (Scholz *et al*. 2011). By establishing a direct link between non‐point source pollution and the mortality syndrome, our findings set the stage for future indicator studies in western North America. This includes, for example, more refined predictive mapping of vulnerable habitats as a function of impervious land cover, at present and with future urban growth scenarios (Feist *et al*. 2011). Coho survival in urban streams can also indicate the success of pollution control programmes, via GSI or other strategies. Intensive control measures will almost certainly be necessary, across large spatial scales, to: (i) recover viable coho populations in the built environment, and (ii) prevent the rapid future loss of coho as a consequence of expanding impervious cover in watersheds that are currently productive but primarily non‐urban.

In the future, it may be possible to narrow the focal list of chemicals by determining more precisely why storm water‐exposed coho are dying. The gaping, surface swimming and disequilibrium of affected spawners suggest adverse physiological impacts on the gill, the heart, the nervous system or some combination of these. An earlier forensic study found no evidence of physical injury to the gills or other tissues (Scholz *et al*. 2011). An alternative approach would be to screen the target organs of symptomatic fish for changes in gene expression, and specifically gene sets that are diagnostic for specific categories of physiological stress (e.g. respiratory uncoupling). If the cause of death is ultimately found to be heart failure, for example, the candidate chemicals could be screened for cardiotoxic potential. It may also be possible to develop alternative exposure methods that reflect different sources of contaminants on roadways. This includes, for example, large‐volume suspensions of particulate soot from motor vehicle exhaust, dust from brake pad wear or fine particles from tyre wear.

Lastly, toxic run‐off is likely to represent an increasingly important conservation challenge for west coast coho populations in the coming years. Extant population segments are generally at historically low abundances, as evidenced by current US Endangered Species Act threatened designations in central and northern California, as well as north‐western Oregon and south‐western Washington. Land cover change has been extensive in some lowland watersheds where coho spawn, as a consequence of sprawl in recent decades (e.g. Robinson, Newell & Marzluff 2005). Over a similar period of time, coho habitat use in areas affected by urbanization has declined sharply (Bilby & Mollot 2008). Resource managers have been aware of the urban pre‐spawn mortality syndrome among adult coho since at least the 1980s (Kendra & Willms 1990). However, the extent to which recurring adult die‐offs have driven down wild coho numbers in urbanizing watersheds is not presently known. Initial modelling has shown that local populations in urbanizing watersheds cannot withstand the rates of mortality observed in Puget Sound urban stream surveys since 2000 (Spromberg & Scholz 2011). However, in terms of recovery planning, this storm water‐related threat has yet to be mapped out for actual coho conservation units at the sub‐basin scale.

In conclusion, a core objective of GSI is to slow, spread and infiltrate storm water. As anticipated from recent studies (e.g. McIntyre *et al*. 2015), the experimental soil columns used here effectively prevented the acutely lethal toxicity of run‐off from a dense urban arterial road. This extends the range of aquatic species and life stages that demonstrably benefit from storm water bioinfiltration. These include the early life stages of zebrafish (McIntyre *et al*. 2014), juvenile coho salmon and their macroinvertebrate prey (McIntyre *et al*. 2015), and adult coho spawners (this study). Bioretention is therefore a promising clean water technology from the standpoint of installation cost, reliability, reproducibility and scalability. However, the science of GSI effectiveness is still relatively young (Ahiablame, Engel & Chaubey 2012), and fundamental questions remain as‐yet unanswered, for example how much treatment will be needed, over what spatial scales, to ensure coho salmon survival? Whereas bioretention may work well for small‐footprint sites that receive modest inputs of storm water, they are but one of many evolving non‐point source pollution control and prevention methods that are currently under development (Hughes *et al*. 2014). For the urban watersheds of the future, the coexistence of humans and wild coho will likely hinge on the success of these innovations.

## Data accessibility

Data generated from this study are included in the text, tables, figures and uploaded online supporting information.

## Supporting information


**Table S1**. Nominal concentrations (μg L^−1^) for metals and selected polycyclic aromatic hydrocarbons (PAHs) in the PAHs/metals mixture and the metals‐only mixture exposures.

**Table S2**. Measured conventional water chemistry parameters in treatments used in adult coho experiments during 2012–2013.

**Table S3**. Measured metal concentrations in treatments used in adult coho experiments during 2012–2013.

**Table S4**. Abbreviations and polycyclic aromatic hydrocarbon (PAH) analytes, including sums of alkyl PAH isomers measured in water samples.

**Table S5**. Measured parent and alkylated homologue polycyclic aromatic hydrocarbons (PAHs) (μg L^−1^) in treatments used in adult coho experiments during 2012–2013.

**Video S1**. Video 1 of a symptomatic adult coho spawner in a Seattle‐area urban stream.

**Video S2.** Video 2 of a field observation of a symptomatic adult coho in a Seattle‐area urban stream.

**Video S3**. Adult coho spawners exposed under controlled experimental conditions to either clean well water, unfiltered urban runoff, or run‐off treated using bioinfiltration.

## References

[jpe12534-bib-0001] Ahiablame, L.M. , Engel, B.A. & Chaubey, I. (2012) Effectiveness of low impact development practices: literature review and suggestions for future research. Water Air and Soil Pollution, 223, 4253–4273.

[jpe12534-bib-0003] Bilby, R.E. & Mollot, L.A. (2008) Effect of changing land use patterns on the distribution of coho salmon (*Oncorhynchus kisutch*) in the Puget Sound region. Canadian Journal of Fisheries and Aquatic Sciences, 65, 2138–2148.

[jpe12534-bib-0004] Brette, F. , Machado, B. , Cros, C. , Incardona, J.P. , Scholz, N.L. & Block, B.A. (2014) Crude oil impairs excitation‐contraction coupling in fish. Science, 343, 772–776. 24531969 10.1126/science.1242747

[jpe12534-bib-0005] Feist, B.E. , Buhle, E.R. , Arnold, P. , Davis, J.W. & Scholz, N.L. (2011) Landscape ecotoxicology of coho salmon spawner mortality in urban streams. PLoS One, 6, e23424. 21858112 10.1371/journal.pone.0023424 PMC3157375

[jpe12534-bib-0006] Gobel, P. , Dierkes, C. & Coldewey, W.C. (2007) Storm water runoff concentration matrix for urban areas. Journal of Contaminant Hydrology, 91, 26–42. 17174006 10.1016/j.jconhyd.2006.08.008

[jpe12534-bib-0007] Heintz, R.A. , Rice, S.D. , Wertheimer, A.C. , Bradshaw, R.F. , Thrower, F.P. , Joyce, J.E. & Short, J.W. (2000) Delayed effects on growth and marine survival of pink salmon *Oncorhynchus gorbuscha* after exposure to crude oil during embryonic development. Marine Ecology Progress Series, 208, 205–216.

[jpe12534-bib-0008] Hughes, R.M. , Dunham, S. , Maas‐Hebner, K.G. , Yeakley, J.A. , Harte, M. , Molina, N. , Shock, C.C. & Kaczynski, V.W. (2014) A review of urban water body challenges and approaches: (2) mitigating effects of future urbanization. Fisheries, 39, 30–40.

[jpe12534-bib-0009] Incardona, J.P. , Carls, M.G. , Day, H.L. , Sloan, C.A. , Bolton, J.L. , Collier, T.K. & Scholz, N.L. (2009) Cardiac arrhythmia is the primary response of embryonic Pacific herring (*Clupea pallasi*) exposed to crude oil during weathering. Environmental Science & Technology, 43, 201–207. 19209607 10.1021/es802270t

[jpe12534-bib-0010] Incardona, J.P. , Swarts, T.L. , Edmunds, R.C. , Linbo, T.L. , Aquilina‐Beck, A. , Sloan, C.A. , Gardner, L.D. , Block, B.A. & Scholz, N.L. (2013) Exxon Valdez to Deepwater Horizon: comparable toxicity of both crude oils to fish early life stages. Aquatic Toxicology, 142–143, 303–316. 10.1016/j.aquatox.2013.08.011 24080042

[jpe12534-bib-0011] Karr, J.R. (1999) Defining and measuring river health. Freshwater Biology, 41, 221–234.

[jpe12534-bib-0012] Kayhanian, M. , Fruchtman, B.D. , Gulliver, J.S. , Montanaro, C. , Ranieri, E. & Wuertz, S. (2012) Review of highway runoff characteristics: comparative analysis and universal implications. Water Research, 46, 6609–6624. 22959661 10.1016/j.watres.2012.07.026

[jpe12534-bib-0013] Kendra, W. & Willms, R. (1990) Recurrent coho salmon mortality at Maritime Heritage Fish Hatchery, Bellingham: a synthesis of data collected from 1987–1989, 28 pp. Washington State Department of Ecology Report 90‐e54, Olympia, Washington, USA.

[jpe12534-bib-0014] King, K.A. , Grue, C.E. , Grassley, J.M. & Fisk, R.J. (2013) Pesticides in urban streams and early life stages of Pacific coho salmon. Environmental Toxicology and Chemistry, 32, 920–931. 23297254 10.1002/etc.2117

[jpe12534-bib-0015] Krahn, M.M. , Rhodes, L.D. , Myers, M.S. , Moore, L.K. , MacLeod, W.D. Jr & Malins, D.C. (1986) Associations between metabolites of aromatic compounds in bile and the occurrence of hepatic lesions in English sole (*Parophrys vetulus*) from Puget Sound, Washington. Archives of Environmental Contamination and Toxicology, 15, 61–67. 3947138 10.1007/BF01055249

[jpe12534-bib-0016] McIntyre, J.K. , Baldwin, D.H. , Beauchamp, D.A. & Scholz, N.L. (2012) Low‐level copper exposures increase visibility and vulnerability of juvenile coho salmon to cutthroat trout predators. Ecological Applications, 22, 1460–1471. 22908706 10.1890/11-2001.1

[jpe12534-bib-0017] McIntyre, J.K. , Davis, J.W. , Incardona, J.P. , Anulacion, B.F. , Stark, J.D. & Scholz, N.L. (2014) Zebrafish and clean water technology: assessing soil bioretention as a protective treatment for toxic urban runoff. Science of the Total Environment, 500–501, 173–180. 10.1016/j.scitotenv.2014.08.066 25217993

[jpe12534-bib-0018] McIntyre, J.K. , Davis, J.W. , Hinman, C. , Macneale, K.H. , Anulacion, B.F. , Scholz, N.L. & Stark, J.D. (2015) Soil bioretention protects juvenile salmon and their prey from the toxic impacts of urban stormwater runoff. Chemosphere, 132, 213–219. 25576131 10.1016/j.chemosphere.2014.12.052

[jpe12534-bib-0019] Meador, J.P. , Sommers, F.C. , Ylitalo, G.M. & Sloan, C.A. (2006) Altered growth and related physiological responses in juvenile Chinook salmon (*Oncorhynchus tshawytscha*) from dietary exposure to polycyclic aromatic hydrocarbons (PAHs). Canadian Journal of Fisheries and Aquatic Sciences, 63, 2364–2376.

[jpe12534-bib-0020] Niyogi, S. & Wood, C.M. (2004) Biotic ligand model, a flexible tool for developing site‐specific water quality guidelines for metals. Environmental Science and Technology, 38, 6177–6192. 15597870 10.1021/es0496524

[jpe12534-bib-0021] van der Oost, R. , Beyer, J. & Vermeulen, N.P.E. (2003) Fish bioaccumulation and biomarkers in environmental risk assessment: a review. Environmental Toxicology and Pharmacology, 13, 57–149. 21782649 10.1016/s1382-6689(02)00126-6

[jpe12534-bib-0022] Pess, G.R. , Montgomery, D.R. , Bilby, R.E. , Steel, E.A. , Feist, B.E. & Greenberg, H.M. (2002) Landscape characteristics, land use, and coho salmon (*Oncorhynchus kisutch*) abundance, Snohomish River, Washington, USA. Canadian Journal of Fisheries and Aquatic Sciences, 59, 613–623.

[jpe12534-bib-0023] Robinson, L. , Newell, J.P. & Marzluff, J.M. (2005) Twenty‐five years of sprawl in the Seattle region: growth management responses and implications for conservation. Landscape and Urban Planning, 71, 51–72.

[jpe12534-bib-0024] Ross, P.S. , Kennedy, C.J. , Shelley, L.K. , Patterson, D.A. , Fairchild, W.L. & Macdonald, R.W. (2013) The trouble with salmon: relating pollutant exposure to toxic effect in species with transformational life histories and lengthy migrations. Canadian Journal of Fisheries and Aquatic Sciences, 70, 1252–1264.

[jpe12534-bib-0025] Sandahl, J.F. , Baldwin, D.H. , Jenkins, J.J. & Scholz, N.L. (2007) A sensory system at the interface between urban stormwater runoff and salmon survival. Environmental Science and Technology, 41, 2998–3004. 17533870 10.1021/es062287r

[jpe12534-bib-0026] Scholz, N.L. , Myers, M.S. , McCarthy, S.G. , Labenia, J.S. , McIntyre, J.K. , Ylitalo, G.M. *et al* (2011) Recurrent die‐offs of adult coho salmon returning to spawn in Puget Sound lowland urban streams. PLoS One, 6, e28013. 22194802 10.1371/journal.pone.0028013 PMC3237429

[jpe12534-bib-0027] Seattle Public Utilities . (2007) City of Seattle State of the Waters 2007: Volume I Seattle Watercourses, 240 pp. Seattle Public Utilities, Seattle, WA.

[jpe12534-bib-0028] da Silva, D.A. , Buzitis, J. , Krahn, M.M. , Bicego, M.C. & Pires‐Vanin, A.S. (2006) Metabolites in bile of fish from Sao Sebastiao Channel, Sao Paulo, Brazil as biomarkers of exposure to petrogenic polycyclic aromatic compounds. Marine Pollution Bulletin, 52, 175–183. 16216283 10.1016/j.marpolbul.2005.08.016

[jpe12534-bib-0029] Sloan, C.A. , Anulacion, B.F. , Baugh, K.A. , Bolton, J.L. , Boyd, D. , Boyer, R.H. *et al* (2014) Northwest Fisheries Science Center's Analyses of Tissue, Sediment, and Water Samples for Organic Contaminants by Gas Chromatography/Mass Spectrometry and Analyses of Tissue for Lipid Classes by Thin Layer Chromatography/Flame Ionization Detection, 61 pp. U.S. Dept. Commerce, NOAA Tech. Memo NMFS‐NWFSC‐125, Seattle, Washington, USA.

[jpe12534-bib-0030] Spromberg, J.A. & Scholz, N.L. (2011) Estimating the future decline of wild coho salmon populations due to early spawner die‐offs in urbanizing watersheds of the Pacific Northwest. Integrated Environmental Assessment and Management, 7, 648–656. 21786416 10.1002/ieam.219

[jpe12534-bib-0031] Stein, E.D. , Tiefenthaler, L.L. & Schiff, K. (2006) Watershed‐based sources of polycyclic aromatic hydrocarbons in urban storm water. Environmental Toxicology and Chemistry, 25, 373–385. 16519297 10.1897/05-285r.1

[jpe12534-bib-0032] Tiefenthaler, L.L. , Stein, E.D. & Schiff, K.C. (2008) Watershed and land use‐based sources of trace metals in urban storm water. Environmental Toxicology and Chemistry, 27, 277–287. 18348619 10.1897/07-126R.1

[jpe12534-bib-0033] Walsh, C.J. , Roy, A.H. , Feminella, J.W. , Cottingham, P.D. , Groffman, P.M. & Morgan, R.P. II (2005) The urban stream syndrome: current knowledge and the search for a cure. Journal of the North American Benthological Society, 24, 706–723.

[jpe12534-bib-0034] Waples, R.S. (1991) Pacific salmon, *Oncorhynchus* spp., and the definition of “species” under the Endangered Species Act. Marine Fisheries Review, 53, 11–22.

[jpe12534-bib-0035] Washington Department of Ecology (2012) Stormwater Management Manual for Western Washington Volume V: Runoff Treatment BMPs. Publication No. 12‐10‐030, 301 pp. Washington Department of Ecology, Olympia, Washington, USA.

[jpe12534-bib-0036] Washington State Department of Transportation (WA DOT) (2013a) 2012 Annual Traffic Report, 231 pp. WA DOT, Olympia, Washington, USA.

[jpe12534-bib-0037] Washington State Department of Transportation (WA DOT) (2013b) State Highway Log Planning Report 2012: SR2 to SR 971, 1781 pp. WA DOT, Olympia, Washington, USA.

