# Supplementary material for: Coho salmon spawner mortality in western US urban watersheds: bioinfiltration prevents lethal storm water impacts
Source: J Appl Ecol. 2015 Oct 8;53(2):398–407. doi: 10.1111/1365-2664.12534 (PMC5019255; doi:10.1111/1365-2664.12534)
Supplement: Supplementary file 1 — Table S1. Nominal concentrations (μg L−1) for metals and selected polycyclic aromatic hydrocarbons (PAHs) in the PAHs/metals mixture and the metals‐only mixture exposures. [file JPE-53-398-s001.docx]

Table S1. Nominal concentrations (ug L^-1^) for metals and selected PAHs in the PAHs/metals mixture and the metals-only mixture exposures.

| Mixture | Contaminant | Low | High |
| --- | --- | --- | --- |
| PAHs/metals | Phenanthrene |  | 0.384 |
|  | Pyrene |  | 0.584 |
|  | Fluoranthene |  | 0.584 |
|  | Cd |  | 1.8 |
|  | Cu |  | 42.0 |
|  | Pb |  | 6.0 |
|  | Ni |  | 12.0 |
|  | Zn |  | 54.0 |
| Metals-only | Cd | 1.5 | 3.0 |
|  | Cu | 50 | 100 |
|  | Pb | 10 | 20 |
|  | Ni | 20 | 40 |
|  | Zn | 150 | 300 |
