# Supplementary material for: Coho salmon spawner mortality in western US urban watersheds: bioinfiltration prevents lethal storm water impacts
Source: J Appl Ecol. 2015 Oct 8;53(2):398–407. doi: 10.1111/1365-2664.12534 (PMC5019255; doi:10.1111/1365-2664.12534)
Supplement: Supplementary file 2 — Table S2. Measured conventional water chemistry parameters in treatments used in adult coho experiments during 2012–2013. [file JPE-53-398-s002.docx]

Table S2. Measured conventional water chemistry parameters for control water and filtered or unfiltered collected highway runoff used in adult coho experiments during 2012-2013.

|  | Treatment | pH | Alkalinity | TSS | SSC | Ammonia | Nitrate | ortho-P | TP | TOC | DOC | Ca | Mg | Hardness |
| --- | --- | --- | --- | --- | --- | --- | --- | --- | --- | --- | --- | --- | --- | --- |
|  | Units | n.a. | mg CaCO3/L | mg/L | mg/L | mg/L | mg/L | mg P/L | mg P/L | mg/L | mg/L | mg/L | mg/L | mg CaCO3/L |
|  | R.L. 2012 | 0.01 | 1 | 1.1 | n.m. | 0.01 | n.m. | 0.004 | n.m. | 1.5 | 1.5 | 0.05 | 0.05 | 0.05 |
| Date | R.L. >2012 | 0.01 | 1 | 1 | 0.2 | 0.01 | 0.025 | 0.005 | 0.005 | 0.5 | 0.5 | 0.05 | 0.05 | 0.05 |
| 15/10/12 | Control | 7.77 | 92.7 | <1.1 | n.m. | 0.278 | n.m. | 0.242 | n.m. | n.m. | <1.5 | 16.3 | 6.7 | 68 |
| 15/10/12 | Unfiltered | 6.73 | 68.2 | 80.9 | n.m. | 3.02 | n.m. | 0.066 | n.m. | n.m. | 91.6 | 45.3 | 4.02 | 130 |
| 29/10/12 | Control | 7.67 | 94.4 | <1.1 | n.m. | 0.296 | n.m. | 0.238 | n.m. | n.m. | <1.5 | 17.6 | 7.03 | 73 |
| 29/10/12 | Unfiltered | 7.47 | 26.5 | 48.2 | n.m. | 0.749 | n.m. | 0.037 | n.m. | n.m. | 11.8 | 16.2 | 1.65 | 47 |
| 2/11/12 | Control | 7.87 | 93.5 | <1.1 | n.m. | 0.287 | n.m. | 0.245 | n.m. | <1.5 | 1.71 | 17.1 | 6.2 | 70 |
| 2/11/12 | Unfiltered | 7.22 | 32.1 | 58.8 | n.m. | 0.319 | n.m. | 0.246 | n.m. | 8.84 | 5.41 | 12.4 | 0.97 | 35 |
| 14/11/12 | Control | 7.81 | 94.8 | <1.1 | n.m. | 0.309 | n.m. | 0.235 | n.m. | 1.71 | <1.5 | 17.2 | 6.7 | 71 |
| 14/11/12 | Unfiltered | 7.06 | 37.4 | 37.6 | n.m. | 0.862 | n.m. | 0.014 | n.m. | 20.8 | 15.4 | 20.1 | 1.64 | 57 |
| 8/11/13 | Control | 7.01 | 130 | <1 | 0.3 | 0.267 | <0.025 | 0.231 | 0.231 | 1.2 | 1.8 | 27 | 10 | 110 |
| 8/11/13 | Unfiltered | 6.12 | 34 | 23 | 14 | 0.397 | 0.45 | 0.009 | 0.118 | 23 | 14 | 18 | 1.6 | 52 |
| 8/11/13 | Filtered | 6.46 | 42 | 26 | 24 | 0.048 | 9.47 | 0.594 | 0.545 | 26 | 24 | 19 | 7.1 | 77 |
| 18/11/13 | Control | 7.78 | 120 | <1 | 0.2 | 0.266 | <0.025 | 0.206 | 0.209 | 1.5 | 1.5 | 24 | 9.7 | 100 |
| 18/11/13 | Unfiltered | 6.81 | 34 | 220 | 190 | 0.933 | 0.48 | <0.005 | 0.4 | 58 | 8.4 | 14 | 2.4 | 45 |
| 18/11/13 | Filtered | 7.03 | 46 | 220 | 220 | 0.032 | 22.3 | 1.04 | 0.988 | 48 | 30 | 30 | 12 | 120 |

R.L = reporting limit

n.m. = not measured
