# Supplementary material for: Coho salmon spawner mortality in western US urban watersheds: bioinfiltration prevents lethal storm water impacts
Source: J Appl Ecol. 2015 Oct 8;53(2):398–407. doi: 10.1111/1365-2664.12534 (PMC5019255; doi:10.1111/1365-2664.12534)
Supplement: Supplementary file 3 — Table S3. Measured metal concentrations in treatments used in adult coho experiments during 2012–2013. [file JPE-53-398-s003.docx]

Table S3. Measured dissolved and total metal concentration (μg L^-1^) in control waters and filtered or unfiltered collected highway runoff used in adult coho experiments during 2012-2013.

|  |  | Dissolved |  |  |  |  | Total |  |  |  |  |
| --- | --- | --- | --- | --- | --- | --- | --- | --- | --- | --- | --- |
|  |  | Cd | Cu | Pb | Ni | Zn | Cd | Cu | Pb | Ni | Zn |
|  | R.L. 2012 | various | 0.1 | 0.04 | 0.1 | 0.5 | 0.02 | 0.1 | 0.04 | 0.1 | 0.5 |
| Date | R.L. 2013 | 0.025 | 0.1 | 0.05 | 0.05 | 0.5 | 0.025 | 0.1 | 0.05 | 0.05 | 0.5 |
| 15/10/12 | Control | <0.02 | 0.16 | <0.04 | <0.1 | 0.42 | <0.02 | 2.29 | <0.04 | <0.1 | 0.83 |
| 15/10/12 | Unfiltered | 0.355 | 105 | 1.41 | 8.24 | 454 | 0.552 | 153 | 13.1 | 10.5 | 589 |
| 29/10/12 | Control | <0.04 | 0.19 | <0.04 | <0.1 | 3.36 | <0.04 | 0.93 | 0.045 | 1.5 | 4.65 |
| 29/10/12 | Unfiltered | <0.1 | 19.6 | 0.527 | <0.1 | 68.1 | 0.184 | 54.9 | 10.3 | 5.41 | 189 |
| 2/11/12 | Control | <0.02 | 0.11 | <0.04 | <0.1 | 0.4 | <0.02 | 0.29 | <0.04 | <0.1 | 0.94 |
| 2/11/12 | Unfiltered | 0.085 | 16.1 | 0.841 | 1.17 | 91 | 0.204 | 66.1 | 12.2 | 4.18 | 204 |
| 14/11/12 | Control | <0.02 | 0.21 | <0.04 | 0.1 | 1.1 | <0.02 | 0.4 | <0.04 | <.01 | 1.06 |
| 14/11/12 | Unfiltered | 0.133 | 31.3 | 1.03 | 2.08 | 135 | 0.197 | 61.4 | 7.97 | 4.23 | 223 |
| 8/11/13 | Control | <0.025 | 0.35 | 0.052 | 0.22 | 4.59 | <0.025 | 1.76 | 0.114 | 0.2 | 10.7 |
| 8/11/13 | Unfiltered | 0.089 | 24.8 | 0.999 | 7.1 | 238 | 0.158 | 57.5 | 7.38 | 6.29 | 405 |
| 8/11/13 | Filtered | 0.051 | 17.8 | 0.474 | 3.64 | 21.2 | 0.071 | 25.9 | 4.64 | 8.22 | 25.1 |
| 18/11/13 | Control | <0.025 | 0.53 | <0.05 | 0.2 | 5.72 | 0.027 | 0.97 | 0.128 | 0.16 | 8.06 |
| 18/11/13 | Unfiltered | 0.077 | 18.9 | 0.438 | 1.99 | 81.4 | 0.363 | 114 | 33.7 | 9.72 | 341 |
| 18/11/13 | Filtered | 0.044 | 1.54 | 0.067 | 0.54 | 62.3 | 0.209 | 41.7 | 12.2 | 17.8 | 57.6 |

R.L. = reporting limit
