# Supplementary material for: Coho salmon spawner mortality in western US urban watersheds: bioinfiltration prevents lethal storm water impacts
Source: J Appl Ecol. 2015 Oct 8;53(2):398–407. doi: 10.1111/1365-2664.12534 (PMC5019255; doi:10.1111/1365-2664.12534)
Supplement: Supplementary file 4 — Table S4. Abbreviations and polycyclic aromatic hydrocarbon (PAH) analytes, including sums of alkyl PAH isomers measured in water samples. [file JPE-53-398-s004.docx]

Table S4. Abbreviations and Polycyclic Aromatic Hydrocarbon analytes, including sums of alkyl PAH isomers measured in water samples.

| NPH: Naphthalene |
| --- |
| C1NPH: C1-Naphthalenes: sum of 1-methylnaphthalene and 2-methylnaphthalene |
| C2NPH: C2-Naphthalenes: sum of dimethyl- and ethyl-naphthalenes, including 2,6-dimethylnaphthalene^a^ |
| C3NPH: C3-Naphthalenes: sum of trimethyl-, methylethyl- and propyl-naphthalenes, including 2,3,5-trimethylnaphthalene^a^ |
| C4NPH: C4-Naphthalenes: sum of tetramethyl-, dimethylethyl-, diethyl-, methylpropyl- and butyl-naphthalenes  DMP: 1,7-Dimethylphenanthrene |
| MN1:  1-Methyl naphthalene |
| MN2:  2-Methyl naphthalene |
| DMN:  2,6-Dimethylnaphthalene |
| TMN:  2,3,5-Trimethylnaphthalene |
| ACY: Acenaphthylene |
| ACE: Acenaphthene |
| FLU: Fluorene |
| C1FLU: C1-Fluorenes: sum of methyl-fluorenes |
| C2FLU: C2-Fluorenes: sum of dimethyl- and ethyl-fluorenes |
| C3FLU: C3-Fluorenes: sum of trimethyl-, methylethyl- and propyl-fluorenes |
| DBT: Dibenzothiophene |
| C1DBT: C1-Dibenzothiophenes: sum of methyl-dibenzothiophenes |
| C2DBT: C2-Dibenzothiophenes: sum of dimethyl- and ethyl-dibenzothiophenes |
| C3DBT: C3-Dibenzothiophenes: sum of trimethyl-, methylethyl-, and propyl-dibenzothiophenes |
| C4DBT: C4-Dibenzothiophenes: sum of tetramethyl-, dimethylethyl-, diethyl-, methylpropyl- and butyl-dibenzothiophenes |
| PHN: Phenanthrene |
| C1PHN: C1-Phenanthrenes and anthracenes^b^: sum of methyl-phenanthrenes and anthracenes, including  1-methylphenanthrene ^a^, 3-methylphenanthrene and 9-methylphenanthrene |
| C2PHN: C2-Phenanthrenes and anthracenes^b^: sum of dimethyl- and ethyl-phenanthrenes and anthracenes, including 1,7-dimethylphenanthrene^a^ |
| C3PHN: C3-Phenanthrenes and anthracenes^b^: sum of trimethyl-, methylethyl-, and propyl-phenanthrenes and anthracenes |
| C4PHN: C4-Phenanthrenes and anthracenes^b^: sum of tetramethyl-, dimethylethyl-, diethyl-, methylpropyl- and butyl-phenanthrenes and anthracenes  ANT: Anthracene |
| MP1:  1-Methylphenanthrene |
| MP3:  3-Methylphenanthrene |
| MP9:  9-Methylphenanthrene |
| RET: Retene |
| FLA: Fluoranthene |
| PYR: Pyrene |
| C1FLA: C1-Fluoranthenes and pyrenes^b^: sum of methyl-fluoranthenes and pyrenes |
| C2FLA: C2-Fluoranthenes and pyrenes^b^: sum of dimethyl- and ethyl-fluoranthenes and pyrenes |
| C3FLA: C3-Fluoranthenes and pyrenes^b^: sum of trimethyl-, methylethyl-, and propyl-fluoranthenes and pyrenes |
| C4FLA: C4-Fluoranthenes and pyrenes^b^: sum of tetramethyl-, dimethylethyl-, diethyl-, methylpropyl- and butyl-fluoranthenes and pyrenes |
| BAA: Benz[*a*]anthracene |
| CHR: Sum of chrysene and triphenylene^c^ |
| C1CHR: C1-Benz[*a*]anthracenes and chrysenes^b^: sum of methyl-benzanthracenes and chrysenes |
| C2CHR: C2-Benz[*a*]anthracenes and chrysenes^b^: sum of dimethyl- and ethyl-benzanthracenes and chrysenes |
| C3CHR: C3-Benz[*a*]anthracenes and chrysenes^b^: sum of trimethyl-, methylethyl-, and propyl-benzanthracenes and chrysenes |
| C4CHR: C4-Benz[*a*]anthracenes and chrysenes^b^: sum of tetramethyl-, dimethylethyl-, diethyl-, methylpropyl- and butyl-benzanthracenes and chrysenes |
| BBF: Benzo[*b*]fluoranthene |
| BKF: Sum of benzo[*k*]fluoranthene and benzo[*j*]fluoranthene^c^ |
| BEP: Benzo[*e*]pyrene |
| BAP: Benzo[*a*]pyrene |
| PER: Perylene |
| IDP: Indeno[1,2,3-*cd*]pyrene |
| DBA: Sum of dibenz[*a,h*]anthracene and dibenz[*a,c*]anthracene^c^ |
| BZP: Benzo[*ghi*]perylene |

^a^ This alkyl PAH is used to calculate the GC/MS response factor for the quantitation of its group of isomers.

^b^ These analytes are quantitated and reported as the sum of their concentrations because these isomers co-occur, and in some cases coelute, in the same region of the GC/MS chromatogram.

^c^ These analytes are quantitated and reported as the sum of their concentrations because they coelute during GC/MS analysis; the first analyte is present in the calibration standard, whereas the additional analyte is not.
