# Supplementary material for: Coho salmon spawner mortality in western US urban watersheds: bioinfiltration prevents lethal storm water impacts
Source: J Appl Ecol. 2015 Oct 8;53(2):398–407. doi: 10.1111/1365-2664.12534 (PMC5019255; doi:10.1111/1365-2664.12534)
Supplement: Supplementary file 5 — Table S5. Measured parent and alkylated homologue polycyclic aromatic hydrocarbons (PAHs) (μg L−1) in treatments used in adult coho experiments during 2012–2013. [file JPE-53-398-s005.docx]

Table S5. Measured parent and alkylated homologue PAHs (μg L^-1^) in control water and filtered or unfiltered collected highway runoff used in adult coho experiments during 2012-2013. Abbreviations are listed in Table S4.

| Date | 15/10/12 | 15/10/12 | 29/10/12 | 29/10/12 | 2/11/12 | 2/11/12 | 8/11/13 | 8/11/13 | 8/11/13 | 18/11/13 | 18/11/13 | 18/11/13 |
| --- | --- | --- | --- | --- | --- | --- | --- | --- | --- | --- | --- | --- |
| PAH | Control | Unfiltered | Control | Unfiltered | Control | Unfiltered | Control | Unfiltered | Filtered | Control | Unfiltered | Filtered |
| 2-Ring |  |  |  |  |  |  |  |  |  |  |  |  |
| NPH | 0.57 | 0.12 | 0.59 | 0.072 | 0.012 | 0.045 | 0.048 | 0.09 | 0.045 | 0.047 | 0.15 | 0.049 |
| C1NPH | 3.3 | 0.15 | 2 | 0.092 | 0.013 | 0.064 | 0.024 | 0.055 | 0.0092 | 0.024 | 0.12 | 0.01 |
| C2NPH | 0.47 | 0.11 | 0.22 | 0.074 | 0.011 | 0.1 | 0.009 | 0.047 | 0.0097 | 0.011 | 0.12 | 0.012 |
| C3NPH | 0.084 | 0.11 | 0.041 | 0.068 | 0.0091 | 0.12 | 0.0082 | 0.045 | 0.0087 | 0.0082 | 0.13 | 0.01 |
| C4NPH | 0.01 | 0.092 | 0.0057 | 0.055 | 0.003 | 0.075 | 0.0041 | 0.043 | 0.0083 | 0.0078 | 0.12 | 0.01 |
| DMP | n.m. | n.m. | n.m. | n.m. | n.m. | n.m. | < 0.0038 | 0.028 | < 0.0050 | < 0.0044 | 0.096 | < 0.0048 |
| MN1 | n.m. | n.m. | n.m. | n.m. | n.m. | n.m. | 0.0071 | 0.018 | < 0.0051 | 0.0074 | 0.034 | < 0.0050 |
| MN2 | n.m. | n.m. | n.m. | n.m. | n.m. | n.m. | 0.017 | 0.037 | 0.0092 | 0.017 | 0.086 | 0.01 |
| DMN | n.m. | n.m. | n.m. | n.m. | n.m. | n.m. | < 0.0039 | 0.011 | < 0.0050 | < 0.0045 | 0.032 | < 0.0048 |
| TMN | n.m. | n.m. | n.m. | n.m. | n.m. | n.m. | < 0.0034 | < 0.0044 | < 0.0045 | < 0.0040 | 0.0083 | < 0.0043 |
| 3-Ring |  |  |  |  |  |  |  |  |  |  |  |  |
| ACY | 0.0025 | 0.013 | 0.0041 | 0.02 | 0.0017 | 0.012 | 0.011 | 0.025 | 0.011 | 0.01 | 0.042 | 0.013 |
| ACE | 0.93 | 0.036 | 0.57 | 0.0064 | 0.0018 | 0.0036 | < 0.0040 | 0.035 | < 0.0052 | < 0.0046 | 0.013 | < 0.0050 |
| FLU | 0.47 | 0.048 | 0.24 | 0.017 | 0.0032 | 0.011 | < 0.0038 | 0.038 | < 0.0050 | < 0.0044 | 0.027 | < 0.0048 |
| C1FLU | 0.016 | 0.033 | 0.008 | 0.021 | 0.00095 | 0.018 | < 0.0038 | 0.015 | < 0.0050 | < 0.0044 | 0.035 | < 0.0048 |
| C2CLU | 0.0071 | 0.079 | 0.0039 | 0.056 | 0.0012 | 0.055 | < 0.0038 | 0.035 | < 0.0050 | < 0.0044 | 0.12 | < 0.0048 |
| C3FLU | 0.0056 | 0.12 | 0.0038 | 0.1 | < 0.00088 | 0.11 | < 0.0038 | 0.066 | < 0.0050 | < 0.0044 | 0.23 | 0.008 |
| DBT | 0.021 | 0.015 | 0.01 | 0.0089 | < 0.00080 | 0.0066 | < 0.0035 | 0.027 | < 0.0046 | < 0.0040 | 0.018 | < 0.0044 |
| C1DBT | 0.00099 | 0.019 | < 0.00071 | 0.021 | < 0.00088 | 0.018 | < 0.0038 | 0.016 | < 0.0050 | < 0.0044 | 0.044 | < 0.0048 |
| C2DBT | < 0.00066 | 0.05 | < 0.00071 | 0.075 | < 0.00088 | 0.064 | < 0.0038 | 0.043 | < 0.0050 | < 0.0044 | 0.16 | 0.0056 |
| C3DBT | < 0.00066 | 0.089 | < 0.00071 | 0.15 | < 0.00088 | 0.12 | < 0.0038 | 0.075 | < 0.0050 | < 0.0044 | 0.28 | 0.011 |
| C4DBT | < 0.00066 | 0.089 | < 0.00071 | 0.15 | < 0.00088 | 0.12 | < 0.0038 | 0.077 | < 0.0050 | < 0.0044 | 0.28 | 0.013 |
| PHN | 0.16 | 0.2 | 0.088 | 0.14 | 0.019 | 0.088 | 0.03 | 0.49 | 0.031 | 0.016 | 0.29 | 0.029 |
| C1PHN | 0.02 | 0.21 | 0.012 | 0.21 | 0.0046 | 0.18 | 0.0058 | 0.2 | 0.011 | < 0.0044 | 0.48 | 0.023 |
| C2PHN | 0.0076 | 0.28 | 0.0063 | 0.33 | 0.0016 | 0.33 | < 0.0038 | 0.15 | 0.0066 | < 0.0044 | 0.56 | 0.021 |
| C3PHN | 0.0018 | 0.29 | 0.0044 | 0.37 | < 0.00088 | 0.38 | < 0.0038 | 0.22 | 0.012 | < 0.0044 | 0.83 | 0.037 |
| C4PHN | < 0.00066 | 0.2 | 0.0036 | 0.26 | < 0.00088 | 0.26 | < 0.0038 | 0.15 | 0.0078 | < 0.0044 | 0.55 | 0.023 |
| ANT | 0.023 | 0.021 | 0.013 | 0.019 | 0.0038 | 0.014 | 0.0049 | 0.043 | 0.0042 | < 0.0034 | 0.037 | 0.0044 |
| Date | 15/10/12 | 15/10/12 | 29/10/12 | 29/10/12 | 2/11/12 | 2/11/12 | 8/11/13 | 8/11/13 | 8/11/13 | 18/11/13 | 18/11/13 | 18/11/13 |
| PAH | Control | Unfiltered | Control | Unfiltered | Control | Unfiltered | Control | Unfiltered | Filtered | Control | Unfiltered | Filtered |
| MP1 | n.m. | n.m. | n.m. | n.m. | n.m. | n.m. | < 0.0038 | 0.04 | < 0.0050 | < 0.0044 | 0.089 | < 0.0048 |
| MP3 | n.m. | n.m. | n.m. | n.m. | n.m. | n.m. | < 0.0038 | 0.057 | < 0.0050 | < 0.0044 | 0.13 | 0.0061 |
| MP9 | n.m. | n.m. | n.m. | n.m. | n.m. | n.m. | < 0.0038 | 0.04 | < 0.0050 | < 0.0044 | 0.098 | < 0.0048 |
| RET | n.m. | n.m. | n.m. | n.m. | n.m. | n.m. | < 0.0038 | 0.018 | < 0.0050 | < 0.0044 | 0.056 | < 0.0048 |
| 4-Ring |  |  |  |  |  |  |  |  |  |  |  |  |
| PYR | 0.0051 | 0.24 | 0.0065 | 0.33 | 0.0055 | 0.3 | 0.01 | 0.82 | 0.025 | 0.006 | 0.81 | 0.051 |
| FLA | 0.0083 | 0.21 | 0.0093 | 0.21 | 0.008 | 0.19 | 0.013 | 0.9 | 0.025 | 0.0073 | 0.51 | 0.039 |
| C1FLA | 0.0012 | 0.082 | 0.0014 | 0.12 | < 0.00088 | 0.11 | < 0.0038 | 0.18 | 0.0058 | < 0.0044 | 0.26 | 0.016 |
| C2FLA | < 0.00066 | 0.11 | 0.0014 | 0.17 | < 0.00088 | 0.16 | < 0.0038 | 0.11 | 0.0069 | < 0.0044 | 0.36 | 0.023 |
| C3FLA | < 0.00066 | 0.13 | 0.0013 | 0.21 | < 0.00088 | 0.19 | < 0.0038 | 0.12 | 0.0078 | < 0.0044 | 0.44 | 0.028 |
| C4FLA | < 0.00066 | 0.11 | 0.0012 | 0.16 | < 0.00088 | 0.15 | < 0.0038 | 0.097 | 0.0065 | < 0.0044 | 0.37 | 0.023 |
| CHR | 0.00062 | 0.096 | 0.002 | 0.12 | 0.0012 | 0.11 | < 0.0033 | 0.3 | 0.012 | < 0.0039 | 0.29 | 0.029 |
| C1CHR | < 0.00035 | 0.11 | 0.0017 | 0.16 | < 0.00046 | 0.15 | < 0.0019 | 0.13 | 0.0098 | < 0.0023 | 0.19 | 0.026 |
| C2CHR | < 0.00035 | 0.15 | 0.0021 | 0.24 | < 0.00046 | 0.2 | < 0.0019 | 0.16 | 0.013 | < 0.0023 | 0.57 | 0.039 |
| C3CHR | < 0.00035 | 0.13 | 0.0025 | 0.22 | < 0.00046 | 0.18 | < 0.0019 | 0.13 | 0.013 | < 0.0023 | 0.49 | 0.036 |
| C4CHR | < 0.00035 | 0.1 | < 0.00037 | 0.18 | < 0.00046 | 0.14 | < 0.0019 | 0.1 | < 0.0025 | < 0.0023 | 0.37 | 0.021 |
| BAA | < 0.00050 | 0.036 | 0.0011 | 0.059 | 0.0011 | 0.054 | < 0.0028 | 0.17 | 0.0054 | < 0.0034 | 0.11 | 0.013 |
| 5-Ring |  |  |  |  |  |  |  |  |  |  |  |  |
| BBF | < 0.00058 | 0.053 | 0.00067 | 0.091 | 0.00077 | 0.08 | < 0.0033 | 0.24 | 0.0079 | < 0.0039 | 0.2 | 0.019 |
| BKF | < 0.00058 | 0.037 | < 0.00060 | 0.058 | 0.00081 | 0.057 | < 0.0033 | 0.2 | 0.0064 | < 0.0039 | 0.14 | 0.016 |
| PER | < 0.00058 | 0.061 | < 0.00060 | 0.096 | < 0.00076 | 0.087 | < 0.0033 | 0.19 | 0.0075 | < 0.0039 | 0.22 | 0.019 |
| BAP | < 0.00052 | 0.029 | < 0.00054 | 0.052 | < 0.00068 | 0.046 | < 0.0030 | 0.18 | < 0.0039 | < 0.0035 | 0.13 | 0.0095 |
| BEP | 0.0023 | 0.013 | 0.0027 | 0.028 | 0.0022 | 0.021 | < 0.0025 | 0.052 | 0.004 | < 0.0029 | 0.067 | 0.0056 |
| DBA | < 0.00044 | 0.0079 | < 0.00045 | 0.015 | < 0.00057 | 0.014 | < 0.0025 | 0.033 | < 0.0032 | < 0.0029 | 0.033 | < 0.0030 |
| 6-Ring |  |  |  |  |  |  |  |  |  |  |  |  |
| IDP | < 0.00052 | 0.039 | < 0.00054 | 0.077 | < 0.00067 | 0.068 | < 0.0029 | 0.17 | 0.0042 | < 0.0035 | 0.15 | 0.012 |
| BZP | < 0.00052 | 0.066 | 0.001 | 0.099 | 0.002 | 0.092 | < 0.0029 | 0.19 | 0.0076 | < 0.0035 | 0.32 | 0.023 |
| TOTAL | 6.11711 | 4.1839 | 3.85867 | 5.0103 | 0.10753 | 4.5932 | 0.1921 | 6.706 | 0.3415 | 0.1617 | 11.2953 | 0.7432 |
